# Ultra-late pancreatic metastases from renal cell carcinoma after nephrectomy: two case reports and a literature review

**DOI:** 10.1093/jscr/rjag091

**Published:** 2026-03-17

**Authors:** Tao Liu, Zhe Cao, Taiping Zhang

**Affiliations:** General Surgery Department, State Key Laboratory of Complex Severe and Rare Diseases, Peking Union Medical College Hospital, Chinese Academy of Medical Sciences and Peking Union Medical College, No. 1 Shuai Fu Yuan, Wangfujing, Beijing, 100730, China; General Surgery Department, State Key Laboratory of Complex Severe and Rare Diseases, Peking Union Medical College Hospital, Chinese Academy of Medical Sciences and Peking Union Medical College, No. 1 Shuai Fu Yuan, Wangfujing, Beijing, 100730, China; General Surgery Department, State Key Laboratory of Complex Severe and Rare Diseases, Peking Union Medical College Hospital, Chinese Academy of Medical Sciences and Peking Union Medical College, No. 1 Shuai Fu Yuan, Wangfujing, Beijing, 100730, China

**Keywords:** PM-RCC, renal cell carcinoma, metastases, pancreas, metastasectomy

## Abstract

Pancreatic metastases of renal cell carcinoma (PM-RCC) are relatively rare. However, they exhibit unique biological behavior, often presenting as late-onset metastases with a relatively favorable prognosis. This report presents two cases of pancreatic masses detected 16 and 20 years, respectively, after radical nephrectomy for renal cell carcinoma (RCC). Both patients were asymptomatic, with the masses identified during routine health examinations. Each patient successfully underwent robot-assisted distal pancreatectomy with splenectomy. Pathological examination confirmed the diagnosis of PM-RCC in both cases, illustrating the characteristic of ultra-late metastasis in RCC. This underscores the necessity for lifelong follow-up in RCC patients and maintaining a high index of suspicion for metastasis when new pancreatic lesions are identified. Carbonic anhydrase-IX-targeted PET/CT may be helpful as an adjunct for preoperative differential diagnosis. Our findings suggest that an aggressive surgical approach may be considered for patients in whom complete resection appears feasible.

## Introduction

Pancreatic metastatic tumors are relatively rare in clinical practice, accounting for approximately 2%–5% of all pancreatic malignancies [1]. Renal cell carcinoma (RCC) is one of the most common primary cancer types to metastasize to the pancreas. Pancreatic metastases of RCC (PM-RCC) are often characterized on contrast-enhanced CT or MRI as well-defined, hypervascular lesions. These radiological characteristics are highly similar to those of pancreatic neuroendocrine tumors (pNETs), and previous studies have reported a misdiagnosis rate as high as 69.2% [2]. RCC is characterized by a propensity for late metastasis and recurrence, with the most common metastatic sites being the lung, bone, liver, brain, and adrenal glands, while metastasis to the pancreas is relatively rare. In most reported series, the median time from RCC surgery to the development of pancreatic metastasis ranges from 6 to 10 years, although intervals exceeding 15 years have been reported in individual cases. Such recurrences occurring more than 15 years after primary nephrectomy are herein defined as ultra-late pancreatic metastases. This unique pattern of recurrence and metastasis poses a significant challenge for long-term surveillance strategies following RCC surgery.

As demonstrated by the two cases presented in this paper, PM-RCC is often discovered incidentally during health examinations. Compared to patients with metastases to other sites, those with PM-RCC exhibit significantly better survival outcomes, with a 5-year OS ranging from 32% to 92.8% following surgical resection (Table 1). The presence of multiple synchronous metastases in other organs from RCC may limit the benefit of surgical resection for pancreatic metastases; however, the prognosis remains superior to that of metastases to other organs [16]. For PM-RCC, tyrosine kinase inhibitors (TKIs) targeting the VEGF pathway have shown favorable efficacy, effectively controlling the progression of pancreatic metastases, inducing tumor shrinkage, and improving overall survival [17]. They are often combined with surgery as the preferred systemic treatment although their clinical role in PM-RCC remains in the preliminary research stage.

**Table 1:** Summary of selected studies on PM-RCC recently.

Study	Country	N (patients)	DFI (months)	Complications	DFS (5 year)	OS (5 year)
Al-Madhi (2024) [3]	Germany	17	154(12–239)	36%	70%	72%
Hajibandeh (2024) [4]	UK	18	58 (4–372)	43.7%	55.50%	55.60%
Boubaddi (2024) [5]	France	42	121 (6–400)	23.8%	29.60%	92.80%
Riemenschneider (2024) [6]	Denmark	25	95.6(12.0–309.7)	36%	32.30%	83.60%
Cignoli (2022) [7]	Italy	33	96(60–120)	NR	NR	75.10%
Blanco-Fernández (2022) [8]	Spain	116	87.35(1.51–323.55)	14%	35%	83%
Milanetto (2020) [9]	Italy	39	84(7–291)	38.50%	52%	79%
Brozzetti (2020) [10]	Italy	26	156(24–420)	NR	57.70%	50%
Chrom (2018) [11]	Poland	34	84(0–235)	NR	NR	41.20%
Fikatas (2016) [12]	Germany	19	122.4	22.20%	NR	71.40%
Tosoian (2014) [13]	USA	42	134.4(0–336)	42.90%	NR	51.80%
Yuasa (2015) [14]	Japan	20	160.8(50.4–152.4)	NR	NR	78.90%
Schwarz (2014) [15]	France	62	117.6(0–300)	NR	27.00%	32%

DFI, disease-free interval; DFS, disease-free survival; OS, overall survival; NR, not reported.

This report describes two cases of PM-RCC that developed 16 and 20 years, respectively, after radical nephrectomy for RCC. These cases illustrate the potential for ultra-late metastatic recurrence in RCC and underscore several important clinical principles: (i) long-term, potentially lifelong, surveillance is necessary following nephrectomy for RCC; (ii) a high index of suspicion for metastatic disease should be maintained in patients with a history of RCC who present with new pancreatic lesions; and (iii) in the era of TKI therapy, a comprehensive treatment strategy incorporating both surgical intervention and TKI therapy may offer potential benefit for PM-RCC.

## Case presentation

### Case 1

A 70-year-old Chinese male underwent open radical left nephrectomy for left RCC (T3aN0M0, Fuhrman nuclear grade I-II) 16 years ago. The surgery was successful, and postoperative recovery was uneventful. No adjuvant therapy was administered, and the patient underwent regular follow-up examinations. One month prior to presentation, an abdominal contrast-enhanced CT scan revealed a 3.6 cm isodense nodule in the tail of the pancreas, which showed significant enhancement in the arterial phase with well-defined borders, raising suspicion for pNET. A subsequent carbonic anhydrase-IX (CAIX)-targeted PET/CT scan using ^18^F-AlF-NYM005 demonstrated high uptake in the pancreatic tail, suggesting possible metastasis from clear cell RCC. During the course of the disease, the patient reported no discomfort such as abdominal pain, diarrhea, abdominal distension, nausea, vomiting, anorexia, jaundice, fever, or new-onset diabetes.

**Figure 1:**
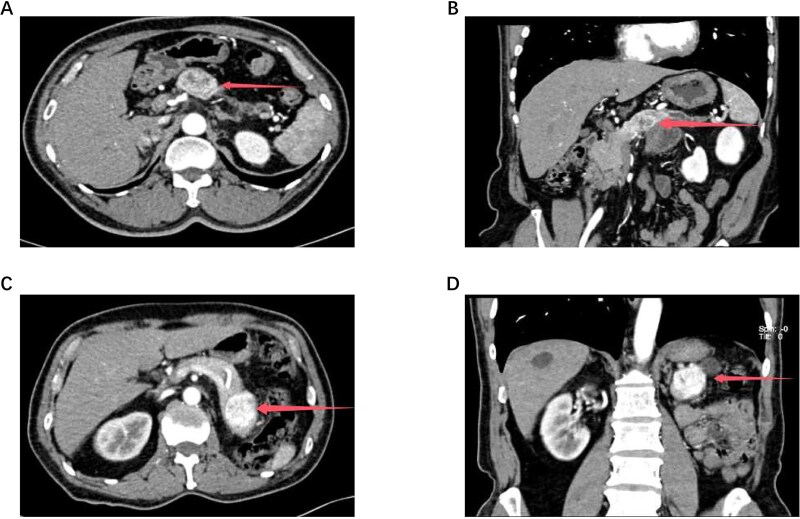
The size and location of the tumors in Cases 1 and 2 are demonstrated by abdominal contrast-enhanced computed tomography (CT) and three-dimensional (3D) reconstruction. Figure 1A shows the tumor in Case 1, and Fig. 1C shows the tumor in Case 2. The corresponding 3D reconstruction results for Cases 1 and 2 are presented in Fig. 1B and Fig. 1D, respectively.

**Figure 2:**
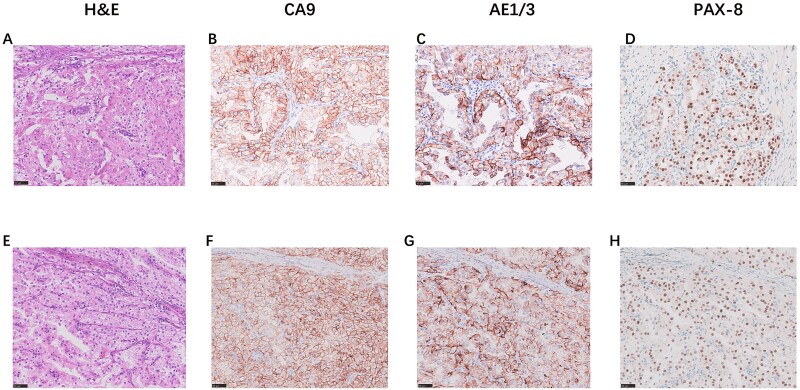
The immunohistochemical staining results for H&E (40x), CA9 (40x), AE1/AE3 (40x), and PAX8 (40x) in Case 1 (Fig. 2A–D) and Case 2 (Fig. 2E–H).

In November 2025, the patient was admitted to our hospital. Tumor marker tests were performed: CA19–9 = 24.9 U/mL, CEA = 3.1 ng/mL, CA242 = 9.4 U/mL, CA125 = 4.2 U/mL, and CA72–4 = 0.8 U/mL. A repeat abdominal contrast-enhanced CT scan indicated a 4.0 cm rounded soft tissue density in the pancreatic tail, with pNET or metastasis considered possible (Fig. 1A–B). After preoperative evaluations ruled out surgical contraindications, the patient underwent robot-assisted radical distal pancreatectomy with splenectomy (KangDuo Surgical Robot-2000 System). The procedure was successful, with no postoperative complications such as hemorrhage or pancreatic fistula. The patient was discharged on postoperative Day 5. The postoperative pathology report confirmed that the pancreatic mass was consistent with metastatic clear cell RCC (WHO Grade 2). Lymph nodes showed chronic inflammation (0/3). An R0 resection (microscopically negative margins) was achieved. Immunohistochemistry results were positive for AE1/AE3, PAX8, CA9, and Vimentin (Fig. 2A–D). The patient is currently under follow-up, receiving adjuvant TKI therapy postoperatively, with no evidence of disease progression to date.

### Case 2

A 61-year-old Chinese male underwent open radical right nephrectomy for right RCC 20 years ago (specific details are not available). The surgery was uneventful, with good postoperative recovery. No adjuvant therapy was administered, and the patient was followed up regularly. Six months before presentation, a routine abdominal ultrasound incidentally detected a mass in the pancreatic body-tail junction, approximately 3.6 cm in size. The patient was asymptomatic, reporting no abdominal pain, distension, diarrhea, jaundice, or new-onset diabetes. One month before presentation, an abdominal contrast-enhanced CT revealed an ill-defined, low-density lesion in the pancreatic body with marked rim enhancement and distal pancreatic atrophy. A subsequent ^18^F-FDG PET/CT showed a metabolically active area in the pancreatic body measuring 5.2 × 2.6 cm with a SUVmax of 4.19, raising suspicion for malignancy.

The patient was admitted to our hospital in January 2024. Tumor marker tests revealed: CA19–9 = 8.6 U/mL, CEA = 2.4 ng/mL, CA242 = 10.9 U/mL, CA125 = 4.6 U/mL, CA72–4 < 1.5 U/mL. An abdominal contrast-enhanced CT confirmed a hyper-enhancing lesion with central necrosis in the pancreatic body, measuring approximately 3.6 × 3.1 cm, suggestive of malignancy (Fig. 1C–D). The lesion was closely related to the splenic artery and vein, with distal pancreatic body and tail atrophy. Subsequently, the patient underwent robot-assisted distal pancreatectomy with splenectomy (KangDuo Surgical Robot-2000 System). The surgery was successful, with an uneventful postoperative recovery and no major complications. The patient was discharged on postoperative Day 7. The postoperative pathology report identified a clear cell tumor within the pancreas, consistent with metastatic renal RCC. Lymph nodes showed chronic inflammation (0/6). Immunohistochemistry was positive for AE1/AE3, PAX8, CA9, and Vimentin (Fig. 2E–H). The surgery achieved a standard R0 resection. The patient has remained recurrence-free for 24 months postoperatively while on TKI therapy.

## Discussion

The prominent features of PM-RCC include a long latency period for metastasis, relatively indolent biological behavior, and favorable long-term prognosis following treatment. The molecular mechanisms underlying late metastasis of RCC remain incompletely elucidated. However, both late-metastatic RCC and PM-RCC share a distinct set of molecular features: frequent PBRM1 loss (60–65%) with retained BAP1 expression. The status of BAP1/PBRM1/SETD2 and the presence of tumor necrosis are independent predictors of late metastasis [18]. Furthermore, PM-RCC exhibits functional enrichment in fatty acid oxidation and angiogenesis pathways, which explains its sensitivity to TKI therapy [19]. Histologically, PM-RCC is characterized by low-grade features, including Fuhrman grade 1–2 nuclei, conspicuous thin-walled vascular networks, nested or tubulopapillary architecture, minimal tumor necrosis, and sparse inflammatory response. These morphologic characteristics correlate with its indolent clinical behavior and excellent prognosis [16, 18].

The imaging manifestations of PM-RCC can be readily misdiagnosed as pNETs due to overlapping features. In Case 1, CAIX-targeted PET/CT provided a valuable diagnostic tool for identifying PM-RCC [20]. Furthermore, when a clear cell-like tumor is identified within the pancreas, histopathologic differential diagnoses should include PM-RCC, clear cell variant of pNET, and serous cystadenoma with clear cell features, in addition to other primary pancreatic clear cell neoplasms.

Unlike most metastatic malignancies, PM-RCC is one of the few scenarios where surgical resection can provide long-term survival benefits. Zerbi *et al*. [21] demonstrated that patients with PM-RCC who underwent surgical treatment exhibited a significantly higher 5-year OS compared to those managed non-operatively (88% vs. 47%). Our literature review indicates that the prognosis following resection is favorable, with reported 5-year OS ranging from approximately 32% to 92.8% and 5-year DFS rates ranging from approximately 27% to 70% (Table 1). The risk of recurrence in PM-RCC is significantly associated with a DFI of less than 10 years and a history of extra-pancreatic metastasis [8]. However, pancreatic surgery carries substantial risk. The PANMEKID study reported an overall postoperative complication rate of up to 60.9%, with Clavien-Dindo grade 3 or higher complications occurring in approximately 14% [8]. Other retrospective studies have generally reported higher rates of grade ≥ 3 complications, ranging from 22.2% to 43.7% (Table 1). Consequently, resection provides substantial benefit in rigorously selected patients with oligometastatic disease or predominantly pancreatic involvement and acceptable operative risk.

Due to the rarity of PM-RCC, there are currently no PM-RCC–specific guidelines or high-level evidence-based recommendations to guide its management. The role of postoperative systemic therapy after complete metastasectomy for PM-RCC remains controversial and is supported mainly by retrospective evidence. Evidence consistently indicates that metastasectomy provides a clear survival benefit for mRCC patients classified as favorable-risk by the modified MSKCC or IMDC prognostic models [22]. You *et al*. [24] also suggested that adjuvant TKIs following complete metastasectomy may improve prognosis, whereas Santoni *et al*. [23] reported that surgery did not improve OS in the era of TKI therapy for PM-RCC (103 vs. 86 months, *P* = .201). In our two patients, considering the uncertainty of current evidence, postoperative VEGF-targeted TKI therapy was initiated following multidisciplinary team discussion as a pragmatic strategy to address potential occult micrometastatic disease.

Notably, even after complete surgical resection, PM-RCC still exhibits a considerable recurrence rate. Consequently, the long-term recurrence characteristics of RCC dictate that follow-up should not be terminated at 5–10 years. It is recommended to establish a long-term, imaging-centric follow-up strategy and to evaluate the possibility of re-treatment for patients with recurrent but locally controllable disease. In this context, CAIX-targeted PET/CT may serve as an effective adjunct [20].

## Conclusion

This paper reports two cases of RCC patients who developed ultra-late pancreatic metastases after radical nephrectomy, both without significant symptoms, highlighting the biological characteristics of long-term latency and delayed metastasis in RCC. CAIX-targeted PET/CT may be considered as an adjunct for differential diagnosis in selected patients. The academic value of such cases lies not only in their rarity but also in their clinical implication: even years after radical treatment for RCC, pancreatic space-occupying lesions should include RCC metastasis as a key differential diagnosis. Follow-up strategies should also consider long-term risks, and treatment approaches should be individualized, integrating surgery and systemic therapy in selected patients.
